# Genomic features separating ten strains of *Neorhizobium galegae* with different symbiotic phenotypes

**DOI:** 10.1186/s12864-015-1576-3

**Published:** 2015-05-02

**Authors:** Janina Österman, Seyed Abdollah Mousavi, Patrik Koskinen, Lars Paulin, Kristina Lindström

**Affiliations:** Department of Food and Environmental Sciences, University of Helsinki, Viikinkaari 9, 00790 Helsinki, Finland; Department of Environmental Sciences, University of Helsinki, Viikinkaari 2a, 00790 Helsinki, Finland; Institute of Biotechnology, University of Helsinki, Viikinkaari 9, 00790 Helsinki, Finland

**Keywords:** *Neorhizobium galegae*, Symbiosis, Genome, *rpoN*, *nifQ*, Nitrogen fixation

## Abstract

**Background:**

The symbiotic phenotype of *Neorhizobium galegae*, with strains specifically fixing nitrogen with either *Galega orientalis* or *G. officinalis*, has made it a target in research on determinants of host specificity in nitrogen fixation. The genomic differences between representative strains of the two symbiovars are, however, relatively small. This introduced a need for a dataset representing a larger bacterial population in order to make better conclusions on characteristics typical for a subset of the species. In this study, we produced draft genomes of eight strains of *N. galegae* having different symbiotic phenotypes, both with regard to host specificity and nitrogen fixation efficiency. These genomes were analysed together with the previously published complete genomes of *N. galegae* strains HAMBI 540^T^ and HAMBI 1141.

**Results:**

The results showed that the presence of an additional *rpoN* sigma factor gene in the symbiosis gene region is a characteristic specific to symbiovar orientalis, required for nitrogen fixation. Also the *nifQ* gene was shown to be crucial for functional symbiosis in both symbiovars. Genome-wide analyses identified additional genes characteristic of strains of the same symbiovar and of strains having similar plant growth promoting properties on *Galega orientalis*. Many of these genes are involved in transcriptional regulation or in metabolic functions.

**Conclusions:**

The results of this study confirm that the only symbiosis-related gene that is present in one symbiovar of *N. galegae* but not in the other is an *rpoN* gene. The specific function of this gene remains to be determined, however. New genes that were identified as specific for strains of one symbiovar may be involved in determining host specificity, while others are defined as potential determinant genes for differences in efficiency of nitrogen fixation.

**Electronic supplementary material:**

The online version of this article (doi:10.1186/s12864-015-1576-3) contains supplementary material, which is available to authorized users.

## Background

The nitrogen-fixing soil bacterium *Neorhizobium galegae* has an easily distinguishable phenotype on the host plant species *Galega orientalis* Lam. and *G. officinalis* L. It is the only rhizobial species known to induce root nodules on *Galega* plants so far, making studies of its genomics an attractive area in the field of research on determinants of host specificity and nitrogen fixation efficiency. Although research on nitrogen fixation with *N. galegae* has been conducted since the description of the new species in 1989 [1], the mechanism(s) behind the specific interactions between *Galega* plants and their microsymbiont is still not well understood. The division of *N. galegae* strains into two symbiovars [2] with different phenotypes on the two host plant species brings further challenge into the study of this bacterium. Work has been done on the rhizobial signalling molecules, the Nod factors, of *N. galegae* [3,4], and the function of the rare acetyl substitution has been investigated. However, a clear explanation for the host specificity observed on *Galega* has not been found within the Nod factors. It is obvious that more information is needed both on characteristics distinguishing *N. galegae* from other rhizobial species but also strains within the species having different symbiotic phenotypes, as well as on characteristics of the host plant that may act to discriminate between strains of the same bacterial species.

The complete genomes of two strains of *N. galegae* were recently sequenced to shed some light on the basic genomic features separating *N. galegae* from other rhizobia and its symbiovars from each other [4]. The complete genome sequences of these strains, the type strain HAMBI 540^T^ representing symbiovar (sv.) orientalis and strain HAMBI 1141 representing sv. officinalis, are invaluable to this research but not enough to observe genomic patterns related to the bacterial species or its symbiovars. Therefore, we have produced draft genomes of eight additional strains of *N. galegae*, four strains each of the symbiovars orientalis and officinalis, which combined with the previously sequenced complete genomes make a good representation of the *N. galegae* population. These data also enable a deeper study of the genomic patterns separating the two symbiovars as well as strains showing different nitrogen-fixing capacities, than was previously possible. In this study, data from the eight newly sequenced strains were combined with the whole-genome data of strains HAMBI 540^T^ and HAMBI 1141, and analysed to produce information on genomic characteristics of the species *N. galegae*. Analysis of subgroups within the species, defined by the symbiotic phenotypes observed on *Galega* plants, together with experimental evidence revealed that the sv. orientalis-specific *rpoN2* gene as well as the *nifQ* gene are necessary for nitrogen fixation. Genes possibly related to enhanced plant growth promoting capabilities are also discussed.

## Results

### Symbiosis gene regions are well conserved within the symbiovars

Draft genomes of eight strains of *N. galegae* were produced, generating genomes consisting of between 54 and 148 contigs. The total size of the sequenced genome is between 6 and 7 Mbp for all strains (Table 1), comprising two to four replicons per strain, as predicted by the number of *repABC* operons and preliminary assembly of contigs. The eight new genomes were analysed together with the previously sequenced strains HAMBI 540^T^ and HAMBI 1141 [4], to produce new information on the genomic differences separating strains of the two symbiovars.

**Table 1:** **Genome features and sequence data for** ***N. galegae*** **strains sequenced in this study**

	**sv. officinalis**				**sv. orientalis**			
	**HAMBI 490**	**HAMBI 1145**	**HAMBI 1146**	**HAMBI 1189**	**HAMBI 2427**	**HAMBI 2566**	**HAMBI 2605**	**HAMBI 2610**
**No. of contigs**	130	66	54	60	65	99	148	59
**Coverage**	36	60	47	40	81	53	61	165
**Total size of contigs (bp)**	6315871	6280747	6409219	6000372	6550167	6546417	6858028	6073615
**No. of predicted genes**	6071	6027	6101	5781	6318	6289	6654	5792
**No. of tRNAs identified** ^**a**^	44	44	43	44	43	44	43	44
**No. of rRNA operons**	3	3	3	3	3	3	3	3
**Sample accession number**	ERS526350	ERS526351	ERS526352	ERS526353	ERS526354	ERS526355	ERS526356	ERS526357

^a^The contig containing the rRNA operon always contains three tRNA genes. Since this contig is always present in three copies in each genome, so are the tRNAs contained within this contig, adding six tRNAs to the total number of tRNAs encountered in the genome, making a total of 49 to 50 tRNAs identified per genome.

**Figure 1:**
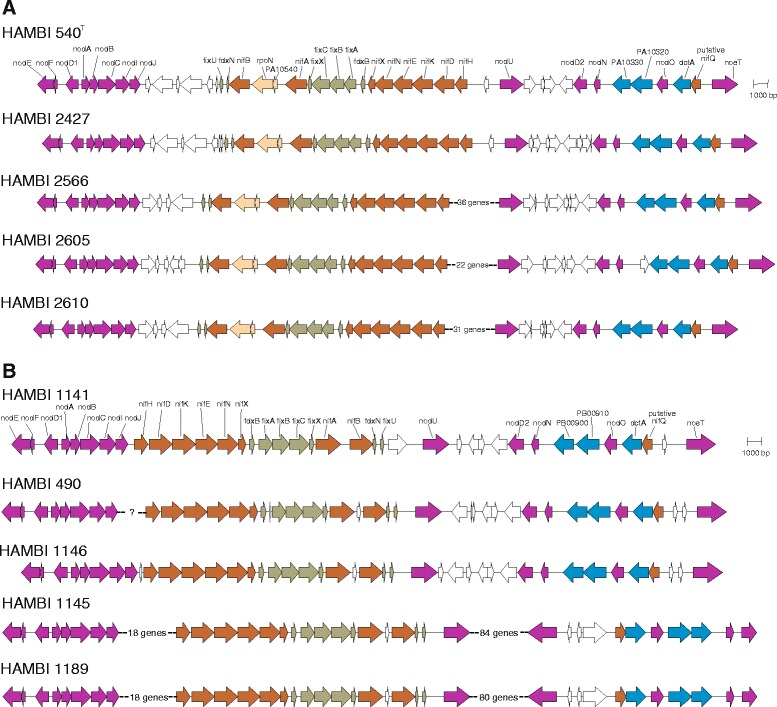
Schematic representation of gene regions containing known symbiosis genes of strains sequenced in this study. Strains HAMBI 540^T^ and HAMBI 1141 are reference strains for which the complete genomes were sequenced previously [4]. **A)** Symbiovar orientalis strains. The genes PA10320 and PA10330 are T1SS genes. **B)** Symbiovar officinalis strains. The genes PB00900 and PB00910 are T1SS genes.

Upon analysis of the *nod*, *noe*, *nif* and *fix* genes found in the gene regions corresponding to the symbiosis gene regions of HAMBI 540^T^ and HAMBI 1141 [4], the gene content was found consistent with regard to the symbiovar (Figure 1). The same *nod*, *noe*, *nif* and *fix* genes can be found in all strains. The four sv. orientalis strains all share the *nod*, *nif* and *fix* gene structure found in strain HAMBI 540^T^, with one exception: a predicted transposase gene located between the gene for the T1SS HlyD family protein and *nodN* in strain HAMBI 2605. Beside this minor difference, the symbiosis gene region only differs between strains in the genes separating the *nodE* – *nodJ* cluster from the *fixU* – *nifH* gene cluster, as well as the genes separating *nifH* from *nodU* and *nodU* from the *nodD2* – *noeT* gene cluster.

The analysed sv. officinalis strains have two main symbiosis gene region structures. Based on preliminary assembly of the contigs included in this region, strains HAMBI 490 and HAMBI 1146 had an almost identical gene content and gene arrangement (although there is no information on the exact sequence between *nodJ* and *nifH* in HAMBI 490), the only difference between the two being a predicted hypothetical protein located in between *fdxB* and *fixA* in HAMBI 490. The symbiosis gene region of these two strains is also very similar to that found in strain HAMBI 1141 (Figure 1). On the other hand, strains HAMBI 1145 and HAMBI 1189 seem to have identical symbiosis gene regions, while differing from strains HAMBI 490 and HAMBI 1146 to some extent. The structure of the main gene clusters is the same, but the genes separating these *nod*, *nif* and *fix* gene clusters differ from those in the latter two strains both in the number of genes present and the predicted function of the same.

### The *rpoN2* gene is specific to sv. orientalis strains

In addition to the *nod*, *noe*, *nif* and *fix* genes, all sv. orientalis strains have a hypothetical protein gene followed by an *rpoN* gene copy included in the symbiosis gene region, similar to the situation in strain HAMBI 540^T^. None of the analysed sv. officinalis strains have these genes. The presence of a second *rpoN* gene in the symbiosis gene region was also confirmed a common characteristic of sv. orientalis strains when a set of nine *N. galegae* sv. orientalis strains (including HAMBI 540^T^, HAMBI 2605 and HAMBI 2610) were screened for this gene by PCR. The RpoN2 protein sequences of all five sequenced *N. galegae* sv. orientalis strains were 100% identical. On the other hand, there were some differences in the RpoN1 sequences of the eight strains where the complete *rpoN1* sequence was available (i.e. all but strains HAMBI 2566 and HAMBI 2605). All eight RpoN1 sequences were between 91.5 and 99.6% identical, while the maximum sequence identity of RpoN2 to any of the RpoN1 sequences was 89.3%.

A gene replacement deletion mutant of the *rpoN2* gene was constructed in HAMBI 540^T^ to test the impact on symbiosis. Location of the insert and intactness of other parts of the genome of the mutant was verified by genome sequencing and mapping to the reference genome of HAMBI 540^T^. Upon inoculation of *G. orientalis* plants with the mutant strain (HAMBI 3480), nodules were formed but the inoculated plants showed growth comparable to that observed when inoculated with the ineffective sv. officinalis strain HAMBI 1141. Plants remained short and the leaves were very pale green, indicating that nitrogen fixation was impaired and that this copy of *rpoN* is required for functional symbiosis on *G. orientalis*.

### The *nifQ* gene is divergent but functional

**Figure 2:**
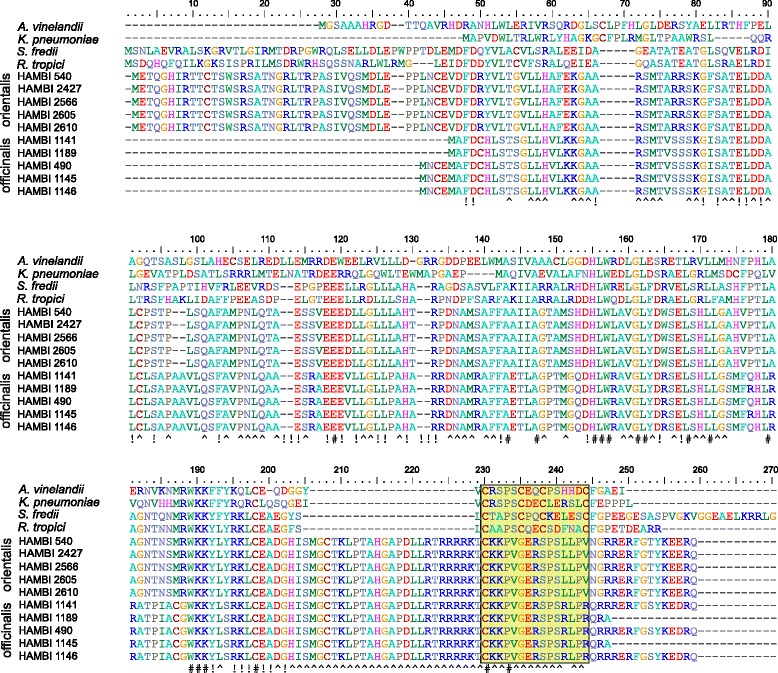
Alignment of NifQ seuquences. The NifQ protein sequences of the ten sequenced *N. galegae* strains were aligned to those of the model system species *A. vinelandii* and *K. pneumoniae*, and the rhizobial relatives *Sinorhizobium fredii* and *Rhizobium tropici*. The last two amino acid residues of *S. fredii* are not visible in the figure. Bottom line: Amino acid residues that are conserved in all *N. galegae* strains are indicated with “^”, residues conserved in all included rhizobial strains with “!” and residues conserved in all strains in the alignment are indicated with “#”. The molybdenum-binding motif region (Cx_4_Cx_2_Cx_5_C [38]), indicated with a yellow box, has not been preserved in *N. galegae*.

Another gene located in the symbiosis gene region that makes a clear distinction between strains of the two symbiovars is the *nifQ* gene. Alignment of the protein sequences deduced from this gene showed that the NifQ protein is well conserved within the symbiovars, while there was a remarkable amount of substitutions when sequences were compared between symbiovars (Figure 2). In addition, the NifQ sequences in *N. galegae* were highly divergent from those of the model species *Azotobacter vinelandii* and *Klebsiella pneumoniae* (Figure 2). The NifQ sequences of all five sv. orientalis strains were 100% identical, while some minor differences in predicted start codon and location of the stop codon could be observed between strains of sv. officinalis.

Gene replacement deletion mutants were constructed also for *nifQ* in both HAMBI 540^T^ and HAMBI 1141, and the mutant strains (HAMBI 3479 and HAMBI 3481 respectively) verified by genome sequencing and mapping to the respective reference strains. The mutant strains were tested on their respective host plants, generating results similar to those observed with the *rpoN2* mutant. Plants were short and pale, clearly suffering from nitrogen deprivation, comparable to the outcome when *G. orientalis* is inoculated with sv. officinalis strain HAMBI 1141 and *G. officinalis* inoculated with sv. orientalis strain HAMBI 540^T^. The nodules formed were small and white on *G. officinalis*, while nodules on *G. orientalis* were more pale pink or greenish, but still much more pale and smaller than effective nodules formed by wild-type HAMBI 540^T^. Unfortunately, attempts to express the *nifQ* gene of HAMBI 1141 in HAMBI 540^T^ and vice versa were not successful. However, these results indicate that even if the *nifQ* genes found in *N. galegae* have diverged from the corresponding genes in related rhizobial species, these are required for nitrogen fixation.

### Secretion systems of type IV and VI are common in *N. galegae*

Because of their interesting ecological functions, the presence of type IV and type VI secretion systems (T4SSs and T6SSs) similar to those previously found in HAMBI 540^T^ and HAMBI 1141 was investigated in the eight newly sequenced strains (Table 2). A T6SS was previously identified in HAMBI 540^T^, while strain HAMBI 1141 has two different types of T4SSs [4]. The two T4SSs in HAMBI 1141 belong to the quorum sensing (QS)-regulated conjugation system (type I) and a type IV conjugation system, based on homology to systems assigned to these types defined by both structural and phylogenetic analyses by Ding and co-workers [5]. Among the three different kinds of secretion systems analysed in *N. galegae*, the T4SS of type I found on the symbiosis plasmid of HAMBI 1141 was the least well represented in the new strains. Although the T4SSs could be identified as being homologs of one of the two systems present in HAMBI 1141, the gene content was not always entirely the same as is found in HAMBI 1141. In HAMBI 1141 there is a *traI*/*traR*/*traM* QS regulation system present on the plasmid pHAMBI1141b together with the T4SS genes of type I. In HAMBI 490, there is what seems to be an incomplete set of QS-related genes located 16 kb from the T4SS of type IV, with two *traR*-like genes and one *traI* homolog but no *traM* gene. In HAMBI 2605, the *traMR* genes are present associated with the T4SS of type I, but no *traI* gene could be found. In addition, the *traACDG* genes are missing. However, the identified type I conjugation system genes in HAMBI 2605 are split onto two different contigs, and thus it is possible that the *traIACDG* genes could not be identified due to a gap in between these contigs. In the same strain there is also a T4SS of type IV with a *virB8*-like gene that is interrupted by a stop codon in the middle of the gene.

**Table 2:** **Type IV and type VI secretion systems in** ***N. galegae*** **genomes**

	**T4SS, type IV** ^**a**^	**T4SS, type I** ^**a**^	**T6SS** ^**a**^
**sv. officinalis strains**			
**HAMBI 1141** ^**b**^	+	+	-
**HAMBI 490**	+	-	-
**HAMBI 1145**	+	-	+
**HAMBI 1146**	+	+	-
**HAMBI 1189**	-	-	+
**sv. orientalis strains**			
**HAMBI 540** ^**T b**^	-	-	+
**HAMBI 2427**	+	-	+
**HAMBI 2566**	-	-	+
**HAMBI 2605**	+	+	+
**HAMBI 2610**	-	-	-

^a^+: similar secretion system found in the strain; − : similar secretion system not found in strain.

^b^strain sequenced previously [4].

T6SS genes were found in strains HAMBI 540^T^, HAMBI 2427, HAMBI 2566, HAMBI 2605, HAMBI 1145 and HAMBI 1189. These strains all have the same T6SS genes arranged in the same gene organisation, the whole gene region showing 83.1-85.0% nucleotide identity compared to HAMBI 540^T^, with the exception of strain HAMBI 2427 which has a nucleotide identity of 99.8%. However, despite the relative abundance of these secretion systems observed in the sequenced *N. galegae* strains, the presence of a certain kind of secretion system could not be linked to strains of either symbiovar.

### Ortholog groups define genes common in specified subgroups

Analysis of ortholog groups was performed on the proteomes of the ten sequenced *N. galegae* strains, to find genes typical for the species and those typical of subgroups of the species. Based on the 4255 ortholog groups shared by all ten strains, the core genome of *N. galegae* comprises between 4323 (HAMBI 1189) and 4346 (HAMBI 540^T^) proteins per strain. The number of strain-specific genes (i.e. singletons in the OrthoMCL analysis) ranges from 111 in HAMBI 2610 to 456 in HAMBI 2605. To investigate whether there are symbiovar-specific genes that could be revealed through analysis of the CDSs of the ten sequenced strains, ortholog groups containing genes from all five strains of one symbiovar, but not a single gene of strains representing the other symbiovar, were targeted. This analysis revealed 40 orientalis-specific ortholog groups and 28 officinalis-specific ortholog groups (Table 3). The officinalis-specific genes are interesting in that all of the 23 genes which are not located on the chromosome of the reference genome, are located either within the symbiosis gene region defined by the *nod*, *nif* and *fix* genes, or within 38 genes downstream of *nodE*. Also among the orientalis-specific genes, 20% are located in the corresponding downstream region of *nodE* in HAMBI 540^T^. In order to investigate a possible connection between the rpoN2 gene and orientalis-specific genes, the putative promoter regions of the orientalis-specific genes were scanned for possible RpoN binding sites in strain HAMBI 540^T^. However, the only possible (although not perfect) motif found was located 238–223 bp upstream of the hypothetical protein gene PA10540.

**Table 3:** **Genes contained in the symbiovar-specific ortholog groups**

**Orientalis-specific genes**		**Officinalis-specific genes**	
**HAMBI 540** ^**T**^ **locus tag**	**Gene function**	**HAMBI 1141 locus tag**	**Gene function**
PA12530	Pimelyl-[acyl-carrier protein] methyl ester esterase	CH10650	Mll9651 protein
PA13300	Drug resistance transporter, EmrB/QacA subfamily	CH10640	RES domain protein
PA12540	Hypothetical protein	CH06450	Hypothetical protein
PA14750	Helix-turn-helix protein, CopG	CH06440	Hypothetical protein
PA10390^c^	Transposase IS4 family protein	PB00510^d^	Hypothetical protein
PA11760	Putative dehydrogenase subunit	PB00490^d^	Hypothetical protein
PA11750	Gluconate 2-dehydrogenase (Acceptor)	PB00470^d^	Benzaldehyde dehydrogenase (NAD+)
PA11740	Transcriptional regulator, LysR family	PB00460^d^	Sigma-54 interacting regulator, V4R domain-containing protein
PA11090	Hypothetical protein	PB00420^d^	Transcriptional regulator
PA11000^a^	Acyl-CoA synthetase	PB00410^d^	Fumarate hydratase
PA10970^a^	Endoribonuclease L-PSP	PB00400^d^	Hypothetical protein
PA10920^a^	Diaminopropionate ammonia-lyase	PB00390^d^	Putative 3-methylaspartate ammonia-lyase, glutamate mutase
PA10900^a^	Hippurate hydrolase	PB00380^d^	3-alpha-hydroxysteroid dehydrogenase
PA10890^a^	Hypothetical protein	PB00370^d^	Steroid C27-monooxygenase
PA10880^a^	Hypothetical protein	PB00360^d^	Agmatinase
PA10870^a^	Hypothetical protein	PB00350^d^	Hypothetical protein
CH26260	Cupin domain protein	PB00340^d^	4-aminobutyrate transaminase
CH26240	Flavodoxin	PB00330^d^	Aldehyde dehydrogenase
PA13080	Transcriptional regulator SocA3	PB00300^d^	Amino acid permease-associated region
PA13090	Glutathione S-transferase	PB00290^d^	Hypothetical protein
CH08120	Carboxymuconolactone decarboxylase	PB00280^d^	Cation/cationic drug transporter
PA04570	Hypothetical protein	PB00270^d^	NADH dehydrogenase
PA10070^b^	Hypothetical protein	PB00260^d^	Transcriptional regulator, TetR family
PA10080^b^	Hypothetical protein	PB00230^d^	MFS transporter
PA14930	Transcriptional regulator, LysR family	PB00220^d^	L-lysine exporter
PA14940	Glyoxalase family protein	PB00210^d^	Transcriptional regulator, ArgP family
PA15010	Hypothetical protein	PB00950^c^	Hypothetical protein
PA10270^b^	Hypothetical protein	CH32360	Hypothetical protein
PA07580	Hypothetical protein		
PA09750	Hypothetical protein		
PA10640^c^	Hypothetical protein		
CH19720	Hypothetical protein		
PA10760^a^	Transposase IS116/IS110/IS902 family protein		
PA10540^c^	Hypothetical protein		
CH11120	Antisigma-factor antagonist, STAS		
CH43990	Hypothetical protein		
PA07480	Putative nitrilase/cyanide hydratase family protein (Carbon-nitrogen hydrolase)		
CH38090	Bacterial regulatory s, tetR family protein		
CH38080	Hypothetical protein		
CH38070	DoxX family protein		

Locus tags of the closed genomes used as gene reference for the homologous genes. Gene functions according to a majority rule where discrepancy is found.

^a^Located within 30 genes downstream from *nodE* in HAMBI 540^T^.

^b^Located within 30 genes downstream from *noeT* in HAMBI 540^T^.

^c^Located within symbiosis gene region.

^d^Located withing 38 genes downstream from *nodE* in HAMBI 1141, i.e. within the extended symbiosis gene region.

Another OrthoMCL analysis was performed comparing the proteomes of HAMBI 540^T^, HAMBI 1141 and eight strains representing closely related rhizobial species (*Rhizobium leguminosarum* sv. viciae, *R. leguminosarum* sv. trifolii, *R. etli*, *R. tropici*, *Sinorhizobium fredii*, *S. medicae*, *S. meliloti* and *Mesorhizobium ciceri*). When the *N. galegae*-specific ortholog groups of this analysis were compared to the core genome of *N. galegae* revealed by the analysis of the ten *N. galegae* strains, finally 441 ortholog groups were found to be common to and specific for all *N. galegae* strains (Additional file 1). Among these, 139 groups consisted of hypothetical proteins, and based on current knowledge none of the remaining ones seem to be directly related to known symbiotic functions.

**Figure 3:**
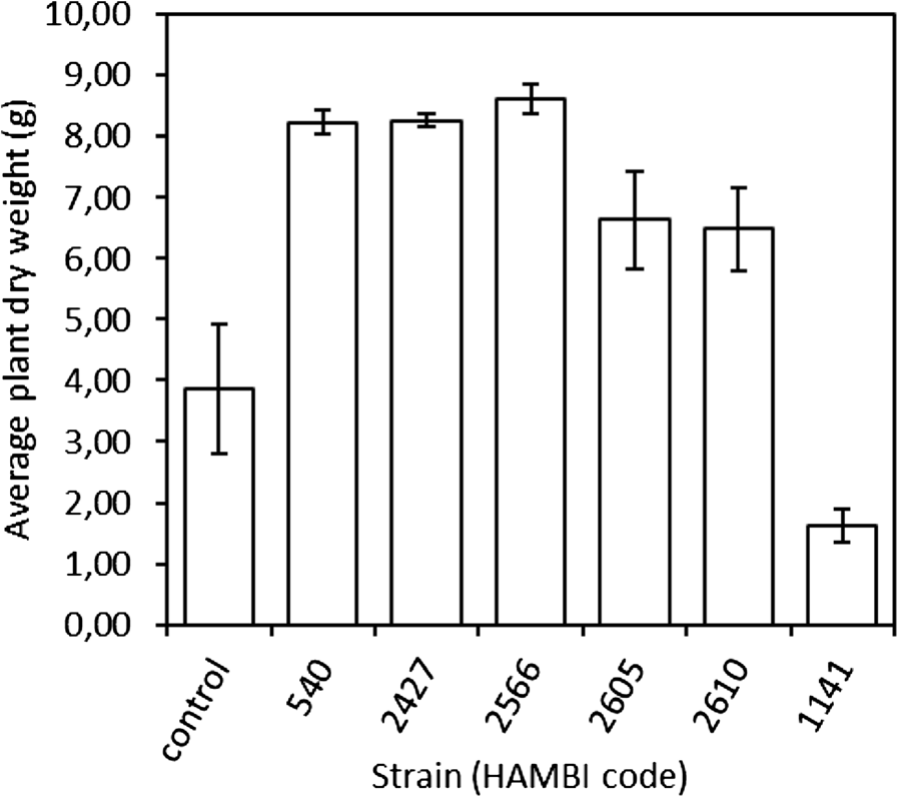
Average dry weight of *G. orientalis* plants inoculated with the genome-sequenced strains. “Control” is the uninoculated control, while HAMBI 1141 is included as a reference for ineffective strains. Dry weight values represent the sum of three plants grown in the same pot. Error bars represent standard error. n = 6 for all strains except for HAMBI 2605 and HAMBI 2610 for which n = 5.

Based on results from a greenhouse experiment testing the plant growth promoting capacity of *N. galegae* strains, the sv. orientalis strains HAMBI 540^T^, HAMBI 2427 and HAMBI 2566 are very good nitrogen fixers (fix^++^), while strains HAMBI 2605 and HAMBI 2610 show a lower level of nitrogen fixation (fix^+^) (Figure 3). To test whether there is a genomics-based pattern that could explain the differences in plant growth promoting efficiency, the OrthoMCL results were analysed from a point of view focusing on the nitrogen fixation properties of the strains. When ortholog groups containing genes shared by the fix^++^ sv. orientalis strains but not present in the fix^+^ strains were analysed, 54 such groups were found (Table 4). Among these groups, 11 were unique to the fix^++^ sv. orientalis strains, i.e. genes not found in any other of the analysed strains but HAMBI 540^T^, HAMBI 2427 and HAMBI 2566.

**Table 4:** **Genes in sv. orientalis fix** ^**++**^ **strains not present in fix** ^**+**^ **strains**

**Gene annotation**	**Locus tag in HAMBI 540** ^**T**^	**Gene annotation**	**Locus tag in HAMBI 540** ^**T**^
Xaa-Pro dipeptidase	PA14880	Nitric-oxide reductase subunit NorB	CH35580
Transposase IS116/IS110/IS902 family protein	PA10750	Nitric-oxide reductase subunit NorC	CH35570
Topology modulation protein	CH30350	Hypothetical protein	CH35560
Hypothetical protein	PA13020	Nitric-oxide reductase NorE protein	CH35550
Hypothetical protein	PA13010	Hypothetical protein	CH35540
PilT protein domain protein	CH43490	NnrS family protein	CH35670
Hypothetical protein	CH43480	Transcriptional regulator NnrR	CH35660
N-methylhydantoinase B	PA14510	Aromatic ring hydroxylating enzyme	CH35650
Hypothetical protein	CH39310	Hypothetical protein	CH35640
Hypothetical protein	PA10050	Transcriptional regulator	PA14580
Hypothetical protein	PA09720	ABC transporter, substrate binding protein (Amino acid)	PA14530
RES domain protein	PA09710	Amino acid ABC transporter, permease protein	PA14540
SH3 type 3 domain-containing protein	PA09040	Amino acid ABC transporter, permease protein	PA14550
Cyanamide hydratase	PA08850	General L-amino acid transport ATP-binding subunit	PA14560
Hypothetical protein	PA05600	Endoribonuclease L-PSP	PA14570
Nitrogen regulatory protein NtrP	PA13210	Phospholipase D/Transphosphatidylase	CH44670
Hypothetical protein	PA08870	Chloride channel protein	PA04790
Hypothetical protein	CH27770	**Lactate dehydrogenase related enzyme**	**PA12720**
Ribosomal protein P2	CH26410	**Aliphatic sulfonates import ATP-binding protein SsuB**	**PA12620**
Glutathionine S-transferase	CH13510	**Sulfonate/nitrate/taurine transport system permease**	**PA12610**
**GntR domain protein**	**PA14430**	**ABC transporter, substrate-binding protein, aliphatic sulfonates family**	**PA12600**
Zinc-binding oxidoreductase	PA01300	**ABC-type nitrate/sulfonate/bicarbonate transport systems periplasmic components-like protein**	**PA12590**
Bacillibactin trilactone hydrolase	PA14790	**Transcriptional regulator GbdR**	**PA12410**
Putative transmembrane protein	CH35630	**Gp49-like PF05973 family protein**	**PA13350**
Amylo-alpha-1,6-glucosidase family protein	CH35620	**DNA-binding helix-turn-helix protein**	**PA13360**
Protein NorD	CH35600	**Chromate transport protein**	**PA08760**
Nitric oxide reductase protein NorQ	CH35590	**Hypothetical protein**	**CH44690**

Annotations according to function assigned to strain HAMBI 540. Genes unique to symbiovar orientalis fix^++^ strains (and thereby not present in any of the sv. officinalis strains) indicated in boldface.

## Discussion

Eight draft genomes of *N. galegae* were produced to enable a more profound study of the bacterial genomics contributing to the host specificity observed in the nitrogen-fixing symbiosis between this bacterium and its host plants. Results of this study show that a major part of the genomes consist of genes common to all analysed *N. galegae* strains, while the two symbiovars can be separated based on a fairly limited set of genes only. The number of strain-specific genes varies a lot between strains, as does the number of replicons present, even if none of the sequenced strains has more than four replicons. Similar conclusions have been drawn for e.g. *S. meliloti* [6] and *R. etli* [7].

### T4SSs and T6SSs are probably not related to symbiosis in *N. galegae*

Type IV and type VI secretion systems in bacteria are important machineries contributing to ecological functions. T4SSs contribute to horizontal gene transfer and might be responsible for transfer of symbiosis genes between strains. In Österman *et al*. 2014 [4] we speculated that the T6SS could play a role in the host specificity of *N. galegae*, being present in HAMBI 540^T^ but not in HAMBI 1141. However, the present study shows that the presence of a certain secretion system can not be attributed to one symbiovar or the other, but these can be found in strains of both symbiovars of *N. galegae*. There are some indications that the T4SSs found in *N. galegae* strains are undergoing changes. The atypical set of QS-related genes in strain HAMBI 490, which has a T4SS classified as type IV (which is not associated with QS regulation [5]), is not necessarily related to regulation of T4S. Moreover, it remains unknown whether the interrupted *virB8*-type gene in HAMBI 2605 renders this T4SS non-functional. The identified T6SS, on the other hand, seems well conserved in the strains of *N. galegae* where such a system is found.

### The specific functions of *rpoN2* and *nifQ* are important for nitrogen fixation

The presence of a gene coding for a hypothetical protein followed by a second *rpoN* gene copy (i.e. another one in addition to the chromosomally located *rpoN*) in the symbiosis gene region appears to be a symbiovar orientalis-specific trait. The absence of amino acid substitutions in the RpoN2 proteins of the analysed strains indicates that the product of this second *rpoN* might perform a very specific function. When compared to the RpoN1 proteins, there is an accumulation of substitutions in the N-terminal region (region I). These differences might affect interactions with target DNA and activator proteins, resulting in transcription of different types of genes as observed in e.g. *Rhodobacter sphaeroides* [8] or transcription under different conditions. Two *rpoN* genes have been found also in other rhizobia, but the reported involvement in symbiosis differs. In *Bradyrhizobium japonicum*, both *rpoN* genes could replace each other functionally, although one of them was regulated in response to oxygen [9]. In *R. etli*, symbiotic nitrogen fixation was drastically reduced when the *rpoN2* gene was mutated, while mutation of the *rpoN1* gene did not affect nitrogen fixation levels [10]. The *rpoN2* gene was not expressed aerobically, but was strongly induced in bacteroids. Also in *Mesorhizobium loti*, the *rpoN2* gene located on the symbiosis island has been shown to be essential for nitrogen fixation, whereas the chromosomally located *rpoN1* gene is dispensable for nitrogen fixation [11]. The results of this study clearly show that the *rpoN2* gene of *N. galegae* is required for nitrogen fixation, but a possible function in determining host specificity should be further investigated. The hypothetical protein gene preceding the *rpoN* gene varies in size between strains, but is nonetheless always part of the same ortholog group. There are, however, no clues to the possible function of this gene.

The *nifQ* gene of *N. galegae* was previously the target of speculations that this gene is non-functional in this species, because of the apparent diversification of this gene from the corresponding gene in related species, as well as the lack of a molybdenum-binding motif [4]. However, this study showed that NifQ is in fact very well conserved within symbiovars, indicating that this could be an important protein after all. Gene replacement deletion studies performed in this work showed that the *nifQ* gene in both symbiovars is required for nitrogen fixation, although the importance of the observed differences in its protein sequence would deserve attention in future studies. Experiments with different levels of molybdenum could have been done to study the effect of the concentration of available molybdenum on the mutant phenotype. However, the results obtained with the conditions used provided enough evidence of the involvement of NifQ in symbiosis, leaving studies on the effect of the molybdenum level on functional symbiosis to the future.

### Analysis of ortholog groups revealed potential future target genes in research on the effectiveness of nitrogen fixation

OrthoMCL analyses allowed the definition of the *N. galegae* core genome as well as a set of genes from the core genome that could not be found in related rhizobial species. Information about the core genome is useful when characteristics of all strains of *N. galegae* are studied, while information on the *N. galegae*-specific portion of the core genome might be useful when studying differences between *N. galegae* and other nitrogen fixers. More specifically, the OrthoMCL analysis of ten *N. galegae* strains revealed symbiovar-specific gene sets, as well as genes present in strains of sv. orientalis known to be good nitrogen fixers while missing in strains known to be less efficient plant growth promoters.

The genes found to be symbiovar-specific are mostly genes involved in transcriptional regulation and metabolic functions. These are not genes that have previously been directly associated with nitrogen fixation, but their possible involvement needs to be investigated in future experiments. In addition, as seen with the *rpoN2* gene located in the symbiosis gene region of sv. orientalis strains, symbiovar-specific gene variants that have high sequence homology with other genes within the genome may not be detected as symbiovar-specific by the OrthoMCL analysis even if these are functionally different and obviously contribute to the pool of genes separating the two groups of strains. The possibility of the orientalis-specific genes being regulated by the orientalis-specific RpoN2 was investigated by searching for known RpoN binding motifs in upstream intergenic regions of these genes. The absence of probable RpoN binding motifs could mean that RpoN2 is either not connected to these genes or it is so specific that it recognises a modified RpoN binding motif compared to the known −24/-12 promoter [12].

Among the genes typical for good *N. galegae* nitrogen fixers in sv. orientalis were the *norEFCBQD* genes, which are all part of the nitric oxide (NO) reductase [13], and the *nnrSR* genes. The *nnr*S gene codes for a haem- and copper-containing membrane protein that is regulated by the product of *nnrR* [14]. These genes were also found as members of the accessory genome relevant for symbiotic interactions in *S. meliloti* [6]. NO production has been observed in functional nodules in bacteroid-containing cells during *Medicago truncatula* – *S. meliloti* symbiosis [15] and has been found to have an important role in stress adaptation and the early stage of *Lotus japonicus* – *M. loti* symbiosis [16]. The *fixLJ* genes are positive regulators of symbiotic expression of *nif* and *fix* genes [17], but also expression of the *nor* genes is dependent on the FixLJ-FixK_2_ regulatory cascade in concert with NO-activated *nnr*R under microaerobic conditions [18,19]. In the light of this information, the presence of NO reduction genes in the efficient nitrogen fixers of *N. galegae* indicates that the possibility to reduce NO might be an advantage for nitrogen fixation.

*B. japonicum* USDA 110 has been found to express genes involved in organic sulphur utilisation in root nodules [20]. The transporter genes for aliphatic sulfonates also found in the sv. orientalis fix^++^-specific gene set are involved in transport of alternative sources of sulphur [21] that can be used for amino acid synthesis. This possibility might be an advantage for strains under stressful conditions.

Another gene found to be present in all superior sv. orientalis nitrogen fixers but none of the less efficient ones, was an *ntrP* gene. The *ntrP* gene is an antitoxin gene forming a toxin-antitoxin (TA) module with *ntrR* in *S. meliloti* [22], found to regulate metabolic processes under stressful conditions such as those encountered when entering symbiosis. The presence of *ntrP*, binding to *ntrR*, lowers the negative effect of *ntrR*, thereby allowing a higher level of expression of genes favourable for nitrogen fixation [22].

## Conclusions

Based on the genomic comparisons performed in this study, differences in genes known to be directly symbiosis-related are small between strains of different symbiovars of *N. galegae*. Nevertheless, the observed symbiovar orientalis-specific *rpoN2* gene as well as the *nifQ* gene, found in both symbiovars, were shown to be important for functional nitrogen fixation. The specific impact of these genes on host specificity should be further investigated. Secretion systems of type IV and type VI are common among strains of *N. galegae*, but do not seem to be involved in symbiotic functions. Based on the functional annotations of genes present in strains known to be good plant growth promoters but not in less efficient ones, an improved ability of nitrogen fixation seems to be correlated with an improved ability to use different metabolic substrates and an optimised regulation of metabolic functions under stressful conditions.

## Methods

### Bacterial strains and growth conditions

*N. galegae* strains used in this study are listed in Table 5. All strains were obtained from the HAMBI culture collection (University of Helsinki, Department of Food and Environmental Sciences, Division of Microbiology and Biotechnology). Strains were grown on TY or YEM agar plates and in TY broth at +28°C.

**Table 5:** **Strains used in this study and applications related to these**

**Strain (HAMBI code)**	**Symbiovar**	**Application** ^a^	**Geographical origin**	**Relevant alternative strain code**	**Reference(s)**
540^T^	orientalis	Gen. seq., PCR	Finland		[1]
2427	orientalis	Gen. seq.	Russia	CIAM 0707	[2,39]
2566	orientalis	Gen. seq.	Caucasus	G058	[40]
2605	orientalis	Gen. seq., PCR	Caucasus	G091	[40]
2610	orientalis	Gen. seq., PCR	Caucasus	G096	[40]
1141	officinalis	Gen. seq.	New Zealand		[1]
490	officinalis	Gen. seq.	Finland		[1]
1145	officinalis	Gen. seq.	New Zealand		[1]
1146	officinalis	Gen. seq.	New Zealand		[1]
1189	officinalis	Gen. seq.	England		[1]
2423	orientalis	PCR	Caucasus	Rg843	[2]
2433	orientalis	PCR	Caucasus	Rg848	[2,39]
2578	orientalis	PCR	Caucasus	G060	[40]
2586	orientalis	PCR	Caucasus	G067	[40]
2609	orientalis	PCR	Caucasus	G094	[40]
2635	orientalis	PCR	Caucasus	G103	[40]

^a^Gen. seq., genome sequencing; PCR, used for PCR screening of *rpoN* in symbiosis gene region.

### DNA isolation

Total DNA of strains used for genome sequencing (Table 5) was isolated using a modified CTAB (hexadecyltrimethylammonium bromide) procedure as described in Österman *et al*. 2014 [4]. DNA for PCR screening was isolated from 6 additional strains of sv. orientalis (Table 5) using one of two different techniques. Most samples were prepared using an UltraClean Microbial DNA Isolation Kit (MO BIO Laboratories, Inc.), but DNA of strains HAMBI 2423 and HAMBI 2433 was prepared using the PrepMan Ultra Sample Preparation Reagent (Life Technologies), applying the protocol for preparation of samples for bacterial and fungal testing from culture broths.

### Screening for rpoN

PCR screening for the second *rpoN* gene originally observed in the symbiosis gene region of HAMBI 540^T^ [4] was performed using primers rpoN-25F (5′-CCGAGTCACACCCAATGTGC-3′) and rpoN-1551R (5′-CGGACGGCCCGGCTATCC-3′) internal to the HAMBI 540^T^ gene. Amplification was done with Phusion High-Fidelity DNA Polymerase (Thermo Scientific) and the HF buffer, using a PCR cycle with initial denaturation at 98°C 30 s, 35 cycles of denaturation 98°C 10 s – annealing 69°C 30 s – elongation 72°C 50 s, and final elongation at 72°C for 10 min. PCR products were verified on a 1% agarose gel. Strains used for screening are listed in Table 5.

### Genome sequencing, assembly and annotation

Genomic DNA (1 μg) was fragmented in a microTube (100 μL) using Covaris S2 (LGC Genomics). Half of the fragmented DNA (50 μL) was purified using a MinElute Reaction Cleanup kit (Qiagen) and eluted in 25 μL EB buffer. End repair and A-tailing was done on the purified DNA (25 μL) using DNA T4 Polymerase (7.5 U), T4 Polynucleotide Kinase (25 U), dNTP (0.2 mM), DreamTaq DNA Polymerase (1.25 U), ATP (5 mM), 2.5 μL T4 Polynucleotide Kinase Buffer A (10x) and 5 μL T4 DNA Polymerase Buffer, in a total volume of 50 μL (all enzymes from Fermentas). The reaction was incubated for 20 min at 25°C, 20 min at 72°C and 10 min at 4°C. The reaction was purified using AMPure XP (Beckman Coulter Inc.) and eluted in 32 μL water. Truncated Illumina forward (5′CTACACTCTTTCCCTACACGACGCTCTTCCGATCT) and complementary reverse (5′Phos-GATCGGAAGAGCACACGTCTGAACTCCAGTCAC) primers were annealed to form a Y-Adapter (2 μL 20 μM) and ligated to the purified end-repaired DNA using 4 μL T4 DNA Ligase buffer (10x), 2 μL T4 DNA Ligase (30 U/μL, Fermentas) in a total volume of 40 μL and incubation for 1 h at 25°C. The ligation reaction was purified using AMPure XP and eluted in 25 μL water. A final PCR was done in a 50 μL reaction using KAPA HiFi DNA Polymerase (5 U), 8 pmol Illumina forward adapter (5′AATGATACGGCGACCACCGAGATCTACACTCTTTCCCTACACGAC), Illumina reverse index primer (5′CAAGCAGAAGACGGCATACGAGATXXXXXXGTGACTGGAGTTCAGACGTGT) and 5 μL purified ligation reaction. The PCR cycle was 95°C for 3 min, 15 cycles of 95°C for 30 s, 60°C for 30 s and 72°C for 1 min, and final extension at 72°C for 5 min. The 6 bp indexes (XXXXXX in reverse index primer above) used were AAGCTA (HAMBI 490), CGTGAT (HAMBI 1145), TTAGGC (HAMBI 1146), GGAACT (HAMBI 1189), ATTATA (HAMBI 2427), GGCCAC (HAMBI 2566), CCGGTG (HAMBI 2605) and GTATAG (HAMBI 2610). The PCR reaction was purified using AMPure XP and eluted in a volume of 20 μL. The library was checked using Bioanalyzer on a DNA High Sensitive chip (Agilent Technologies). The concentration was measured using a High Sensitive kit on Qubit (Invitrogen). The libraries were pooled and sequenced in two partial paired-end runs on an Illumina MiSeq sequencer using the v2 and v3 sequencing kit.

The obtained MiSeq raw sequences from both sequencing rounds were subjected to quality filtering and overlapping sequences with a Phred quality score Q30 or above were extended using FLASH [23] and assembled using Newbler (Roche). Gene prediction was done with Prodigal ver. 2.50 [24] followed by functional annotation with the PANNZER tool [25]. The tRNA genes were annotated using tRNAscan-SE 1.3.1 [26] and rRNA genes identified with RNAmmer 1.2 [27] and alignment to corresponding genes of HAMBI 540^T^ and HAMBI 1141. The gene predictions of known symbiosis-related genes were manually checked. The draft genomes were submitted to the European Nucleotide Archive in the form of contigs [EMBL: ERS526350- ERS526357]. The sequences can be accessed through the link http://www.ebi.ac.uk/ena/data/view/PRJEB6976.

### Mutant construction and verification

Gene replacement deletion mutants of *rpoN2* (∆*rpoN2*::Ω-Km) and *nifQ* (∆*nifQ*::Ω-Km) were constructed by marker exchange where the target gene was replaced with the Ω-Km interposon [28] containing the *nptII* gene. The *rpoN2* gene was mutated in strain HAMBI 540^T^ (mutant strain HAMBI 3480) and the *nifQ* gene in both HAMBI 540^T^ (mutant strain HAMBI 3479) and HAMBI 1141 (mutant strain HAMBI 3481). Upstream flanking regions, the left arms, of the genes to be mutated were amplified (1167 bp for *rpoN2*, primers RpoNLLSpeI and RpoNLRBamHI; 1161 bp for HAMBI 540^T^*nifQ*, primers nifQLLSpeI-ori and nifQLRBamHI-ori; 1092 bp for HAMBI 1141 *nifQ*, primers nifQLLSpeI-2 and nifQLRBamHI-2) as well as downstream flanking fragments, the right arms (1073 bp for *rpoN2*, primers RpoNRLBglII and RpoNRRXhoI; 1092 bp for *nifQ* in both HAMBI 540^T^ and HAMBI 1141, primers nifQRLBamHI and nifQRRXhoI), using Phusion or DyNAzyme II polymerase (Thermo Scientific). Primer sequences are listed in Table 6. The amplified fragments contained short regions of the 5′ and 3′ ends respectively, of the genes. The primers contained restriction endonuclease sites (BamHI and SpeI for the left arm and BglII or BamHI and XhoI for the right arm) to facilitate directional cloning. The Ω-Km interposon was released from pHP45Ω-Km [27] by BamHI digestion, purified and ligated along with the PCR products (digested with BamHI + SpeI and BglII/BamHI + XhoI respectively and purified) into pJQ200SK [29] that had been digested with SpeI and XhoI and dephosphorylated. The resulting constructs where the Ω-Km interposon was inserted between the two PCR products was transferred into *Escherichia coli* S17-1 λpir by electroporation (ca 30 ng of plasmid construct into 40 μL of electrocompetent cells, electroporation at 2.5 kV, 25 μF and 200 Ω in 0.2 cm spaced cuvettes) and confirmed by restriction analysis and sequencing (sequencing primers T3 as well as gene-specific primers rpoNL-646, oriNifQL-570 or offNifQL-661 for the left arm; M13 UP as well as gene-specific primers rpoNR-413, oriNifQR-583 or offNifQR-560 for the right arm). The verified constructs were then transferred into *E. coli* ST18 [30], the donor strain used to transfer each construct into *R. galegae* HAMBI 540^T^ and HAMBI 1141 (*nifQ* only) by biparental spot mating. Mating was conducted by mixing stationary-phase recipient with late log-phase or stationary-phase donor, pelleting the cells, followed by resuspension in 50 μl of MilliQ water and spotting on a TY plate with 5-aminolevulinic acid (ALA; 50 μg/mL). Exconjugants were plated onto def8 agar [31] containing 5% sucrose and neomycin (25 μg /mL), or TY agar containing 5% sucrose and neomycin (50 μg/mL), to select for cells in which the suicide plasmid had been inserted and pJQ200SK removed via recombination events. Mutant candidate clones were colony purified on TY (Nm 50 μg/mL) plates and tested for sensitivity to gentamicin. The final neomycin resistant, gentamicin sensitive gene replacement mutants were further confirmed by PCR analysis and sequencing. The insert-flanking regions were amplified with two sets of primers: rpoNmutLL (*rpoN*), 540nifQL-1234 F (HAMBI 540^T^*nifQ*) or 1141nifQL-1184 F (HAMBI 1141 *nifQ*) together with hsnTmutLR, amplifying from within the *Rhizobium* DNA upstream of the left arm to the 5′ end of the interposon; and primers hsnTmutRL and rpoNmutRR (rpoN) or nifQR-1209R (HAMBI 540^T^ and 1141 *nifQ*), amplifying a fragment from within the 3′ end of the interposon to the *Rhizobium* DNA downstream of the right arm. These PCR fragments were sequenced over the junctions to confirm that homologous recombination had worked properly.

**Table 6:** **Primers used in this study**

**Primer**	**Sequence 5′-3′**
*PCR amplification*	
RpoNLLSpeI	TTTAAAACTAGTTTGATATCGTCACCAATGGC
RpoNLRBamHI	AAATTTGGATCCGTTTTCATTCAGAGCTCCTG
nifQLLSpeI-ori	TTTAAAACTAGTCGATCGTCGTGAGCGCAATG
nifQLRBamHI-ori	AAATTTGGATCCAGTTTCCATCCTGCGACCTC
nifQLLSpeI-2	AAATTTACTAGTGCTGCATCTCGGGAGGTCGAT
nifQLRBamHI-2	TTTAAAGGATCCAGCCATCTCGCAATTCACAGCG
RpoNRLBglII	AAATTTAGATCTTTCTGACTCTGTCATGCTTC
RpoNRRXhoI	TTTAAACTCGAGGTCCATAGTAAGTGCCGTGA
nifQRLBamHI	AAATTTGGATCCCGACAATGATTGCGAATCAAGC
nifQRRXhoI	TTTAAACTCGAGCGGCCGAACCCTTTGAACTC
rpoNmutLL	AAACAAGATCATGCCATCGG
540nifQL-1234F	TAGTCCGCCTGAGTCCAGAG
1141nifQL-1184F	GCTTGTGATTGACGAGGTG
hsnTmutLR	ACTATCAGGTCAAGTCTGCT
hsnTmutRL	TTGATGTTACCCGAGAGCTT
rpoNmutRR	CTTCATCCGATCCTGGAGTA
nifQR-1209R	TCGCTATTGCATTGCCGATT
*Sequencing*	
rpoNL-646	TGCAGTACAGCGATTGTGACG
oriNifQL-570	TGACGCAAGCAGTCGGCAAC
offNifQL-661	CATGGCAACGATGGCAAGTC
rpoNR-413	GCAACCGCAACGTGTATTCG
oriNifQR-583	GGATATTGCCATCCGCGAAG
offNifQR-560	TACCGAGATGAACAGCACCTG

The mutant strains were finally whole-genome sequenced to verify that the insert was present only in the intended location, replacing the deleted gene, and that no other deviations from the reference strain were present. Total DNA of the strains was isolated using the CTAB method as described in section “DNA isolation”. Sequencing was done on an Illumina MiSeq sequencer as described above for the eight genome-sequenced strains, using the v3 sequencing kit, to coverages of 26 x (HAMBI 3480), 23 x (HAMBI 3479) and 24 x (HAMBI 3481). Raw sequence reads were quality filtered and the sequences with a Phred quality score Q25 or above were extended with FLASH [23] and both extended and non-extended fragments assembled using Newbler (Roche). The resulting contigs were then mapped to the reference genome (HAMBI 540^T^ or HAMBI 1141) using the BWA-SW algorithm of the BWA software package [32]. The mapping results were then manually checked for consistency.

### Plant tests of mutants

Nodulation tests of the mutant strains were performed on their respective original hosts, *Galega orientalis* or *Galega officinalis. G. orientalis* seeds were surface sterilised by washing the seeds in 96% ethanol for 1 minute, 3% sodium hypochlorite for 3–5 minutes and washing with sterile water 5–6 times for 1–2 minutes. The sterilised seeds were germinated on TY agar plates at room temperature in darkness. *G. officinalis* seeds were surface sterilised by the following procedure: washed with concentrated sulphuric acid for 15 minutes, rinsed with sterile water 8 times for 2 minutes, kept in 96% ethanol for 1 minute and finally washed with sterile water 6 times for 2–5 minutes. Germinated seeds were transferred to glass jars containing a mixture of Leca gravel (4–10 mm), sand (0.5-1.2 mm) and vermiculite. The components were washed and mixed at a ratio 3:5:5, jars were filled with the mixture and sterilised at 160°C for 24 h. Each jar (containing about 700 mL of soil mixture) was watered with 125 mL quarter-strength nitrogen-free Jensen medium [33] before seeds were planted, three plants per jar. Inoculant strains were grown in TY medium to an OD_600_ of about 1.0, pelleted and resuspended in water, and plant seedlings inoculated with 1 mL bacterial suspension. Negative control seedlings were inoculated with 1 mL of sterile MilliQ water. Inoculated seedlings were covered and each jar watered with 50 mL sterile water. Plants were then grown in a growth chamber (20°C for 1 h, 24°C for 16 h, 20°C for 1 h, 16°C for 6 h with darkness) for four weeks.

### Greenhouse experiment

The bacterial strains used to inoculate *G. orientalis* plants were cultured on yeast mannitol agar (YMA) medium at 28°C for 2 days, followed by culture of single colonies in 50 ml TY medium at 28°C for 48 hours. The bacterial cultures were centrifuged and the pellets resuspended in sterile water (cell density of 1 × 10^8^ cells/mL). *G. orientalis* seeds were sterilized as described above, and sown in pots (3 L, 15 cm high, 19 cm in diameter) filled with a coarse potting mix (Kekkilä) (N-P-K 14-4-20, pH 5.4), 6 seeds per pot. Each seed was inoculated with 1 mL of bacterial suspension (sterile water for the uninoculated control). The plants were grown under climate-controlled greenhouse conditions, applying a randomized complete block design with 6 replicates. The greenhouse conditions were as follows: relative humidity 70% (weeks 0–3) or 40% (weeks 4–7), and a 16-hour photo period with a temperature of 24°C at day and 15°C at night. The number of seedlings per pot was thinned out to 3 seedlings 10 days after inoculation. The plants were harvested after 7 weeks to ensure that the nitrogen content of soil was depleted, and a second harvest was done 42 days after the first harvest. After the second harvest, the aerial parts of the plants were dried at 75°C and dry weights determined as the sum of all plants from the same pot. For strains HAMBI 2605 and HAMBI 2610, growth of one plant in one pot each failed, reducing the number of pots included in dry weight measurements for these strains to 5.

### Bioinformatics analyses

The OrthoMCL software [34] was used to find ortholog groups between the eight newly sequenced strains of *N. galegae* and the two reference strains HAMBI 540^T^ and HAMBI 1141 [4], as well as between HAMBI 540^T^, HAMBI 1141 and eight related rhizobial species: *S. medicae* WSM419, *S. meliloti* 1021, *S. fredii* NGR234, *R. leguminosarum* bv. viciae 3841, *R. leguminosarum* bv. trifolii WSM2304, *R. etli* bv. mimosae str. Mim1, *R. tropici* CIAT 899, *M. ciceri* bv. biserrulae WSM1271. The software was run with default settings. Custom Perl, Python and Biopython [35] scripts were used to modify output from these analyses. Alignment of NifQ proteins (accession numbers of previously published sequences: *A. vinelandii* AAA22151, *K. pneuomonie* WP_032733323, *S. fredii* HH103 AAG37298, *Rhizobium tropici* CIAT 899 AGB73564, HAMBI 540^T^ CDN51705, HAMBI 1141 CDN58442) was done using MUSCLE [36] and manually curated. Prediction of RpoN binding sites was carried out using the TFBS prediction of the online tool PePPER [37]. Both *E. coli* K12 RpoN and *B. japonicum* USDA 110 RpoN1, available in PePPER, were considered when the intergenic regions of the sv. orientalis-specific genes were searched for RpoN binding sites.

### Availability of supporting data

The data sets supporting the results of this article are included within the article and its additional files. The genome sequences of *N. galegae* strains HAMBI 490, HAMBI 1145, HAMBI 1146, HAMBI 1189, HAMBI 2427, HAMBI 2566, HAMBI 2605 and HAMBI 2610 are publicly available as contigs in the European Nucleotide Archive [EMBL:CCRG01000001-CCRG01000130 (HAMBI 490), EMBL:CCRH01000001-CCRH01000066 (HAMBI 1145), EMBL:CCRI01000001-CCRI01000054 (HAMBI 1146), EMBL:CCRK01000001-CCRK01000060 (HAMBI 1189), EMBL:CCRJ01000001-CCRJ01000065 (HAMBI 2427), EMBL:CCRL01000001-CCRL01000099 (HAMBI 2566), EMBL:CCRM01000001-CCRM01000148 (HAMBI 2605), EMBL:CCRN01000001-CCRN01000059 (HAMBI 2610)].

## Additional file

Additional file 1:
**This table lists the functional annotations of genes in the 441 ortholog groups that define** ***N. galegae*** **-specific genes when analysis of the ten** ***N. galegae*** **strains was combined with analysis of the reference genomes HAMBI 540** ^**T**^ **and HAMBI 1141 with eight other rhizobial genomes (** ***S. medicae*** **WSM419,** ***S. meliloti*** **1021,** ***S. fredii*** **NGR234,** ***R. leguminosarum*** **bv. viciae 3841,** ***R. leguminosarum*** **bv. trifolii WSM2304,** ***R. etli*** **bv. mimosae str. Mim1,** ***R. tropici*** **CIAT 899,** ***M. ciceri*** **bv. biserrulae WSM1271).** The genes of strain HAMBI 2427 are given as representatives of the whole ortholog group.

## Abbreviations

sv.: Symbiovar
T4SS: Type IV secretion system
T6SS: Type VI secretion system
QS: Quorum sensing
CDS: Protein-coding DNA sequence
ABC transporter: ATP-binding cassette transporter
NO: Nitric oxide
TA: Toxin-antitoxin
ALA: 5-aminolevulinic acid
TY: Tryptone yeast
YMA: Yeast mannitol agar
